# A Retrospect of the Special Issue “Advances in Oral Implant Health”

**DOI:** 10.3390/dj11040093

**Published:** 2023-03-31

**Authors:** Andrea Butera, Andrea Scribante

**Affiliations:** 1 Unit of Dental Hygiene, Section of Dentistry, Department of Clinical, Surgical, Diagnostic and Pediatric Sciences, University of Pavia, 27100 Pavia, Italy; 2 Unit of Orthodontics and Pediatric Dentistry, Section of Dentistry, Department of Clinical, Surgical, Diagnostic and Pediatric Sciences, University of Pavia, 27100 Pavia, Italy

Research on modern dental implantology focuses on the development of increasingly more advanced techniques with the aim of improving the reliability of dental implants while reducing patient morbidity. The biology of osseointegration has been extensively explored, several chemical and physical implant surface treatments have been proposed to improve osseointegration, and new surgical techniques based on regenerative procedures have been introduced that enable implant positioning, even in cases of severe bone loss. Finally, the introduction of a digital workflow for both surgical and prosthetic planning has significantly changed the approach to clinical practice [1,2,3].

Despite this continuing evolution, several concerns are still related to oral implantology, such as systemic conditions limiting the possibility of recurring implant surgery and the risk of developing peri-implant disease. In order to guarantee the optimal health of the peri-implant tissues; probiotics; ozonized water; lasers; and low particle size powders, such as glycine and erythritol, have been proposed [4,5,6]. This non-surgical peri-implant therapy aims to reduce plaque accumulation at the different implant/mucosal interfaces, particularly at the transition point between the crown and the abutment [7,8].

Based on these considerations, the current Special Issue entitled “Advances in Oral Implant health” had the objective of collecting in vivo studies, case series, case reports, and reviews that could provide new knowledge in the field of implant surgery and the proactive maintenance of implant surfaces and peri-implant tissues [9].

The first research collected in this Special Issue consisted of a clinical study by Van Orten et al. [10] aiming to assess the impact of autologous dentin particles mixed with injectable platelet-rich fibrin (i-PRF) on a sticky tooth mixture for socket preservation in terms of the consecutive need for horizontal guided bone regeneration and histological findings. The authors noticed that this method is valuable for socket preservation and obtaining vital and good-quality bone structure. The sticky tooth technique seems very efficient despite the more complex equipment.

In the study by Cosola et al. [11], the authors performed a radiographic and histomorphologic evaluation of the maxillary bone after a sinus lift using the crestal approach, with subsequent insertion of adsorbable collagen. Histomorphological analyses confirmed the quality of the new bone formation even without graft biomaterials, which could be due to the enlargement of the space, meaning more vascularization and stabilization of the coagulum. Therefore, the authors conclude that using just collagen could be sufficient to promote good new bone formation with minimally invasive surgery.

In the case report by Carossa et al. [12], the 2-year clinical outcome of a full-arch rehabilitation using trans-mucosal tissue-level implants with and without implant-abutment units was presented. In a female patient seeking mandibular rehabilitation, two anterior implants were inserted straight and connected directly to the prosthesis (no abutments), whereas the two distal implants were tilted to avoid the alveolar nerve and connected to two 30° angulated abutments. Two years post-implant placement, all implants were successfully integrated, resulting in an implant survival rate of 100%.

The last research collected in this Special Issue consisted of a literature review aiming to evaluate the use of bioengineering tools, finite element analysis, strain gauge analysis, photoelastic analysis, and digital image correlation in computational studies with greater validity and reproducibility [13]. According to their bibliometric analysis, the authors evidenced a significant variation in the number of articles in the two databases considered. Moreover, they concluded that modern dentistry findings in finite element analysis, strain gauge, photoelastic, and digital image correlation that analyze the biomechanical behavior in dental materials obtain results that support rehabilitations with favorable prognosis and patient satisfaction.

Based on the considerations mentioned above, the authors of the present Special Issue would like to thank all clinicians and researchers who contributed their relevant manuscripts, and would also like to suggest a second volume of the Special Issue (“Advances in Oral Implant Health: Volume II”) for further research on the topic.
